# Discovery and Biological Evaluation of Natural Phenolic Antioxidants

**DOI:** 10.1155/2017/2649129

**Published:** 2017-11-16

**Authors:** Jie Li, Keyvan Dastmalchi, Lin-sen Qing, Pei Luo

**Affiliations:** ^1^University of California, San Diego, San Diego, CA, USA; ^2^The City College of New York, New York, NY, USA; ^3^Chengdu Institute of Biology, Chinese Academy of Sciences, Chengdu, China; ^4^Macau University of Science and Technology, Taipa, Macau

Oxidative stress results from the imbalance between reactive oxygen or nitrogen species and antioxidants and has been increasingly recognized as a significant contributing factor in the pathogenesis of various disorders, such as pulmonary disorders and cardiovascular diseases. Although well known as antioxidants and widely perceived for their beneficial effects, a large number of phenolic natural products remain underexplored and more evidence is needed to elucidate the molecular mechanisms of different classes of phenolic compounds involved in the potential protective effects against oxidative stress. This special issue aimed to highlight the discovery as well as biological evaluation of natural phenolic antioxidants. From about 20 manuscripts received, we selected 7 primary research articles and 2 reviews that addressed different aspects of those objectives.

X. Ren et al. reported that the role of resveratrol, a phenolic antioxidant, in regulating mitochondrial functions and dynamics during the cardiac aging process involves the Drp1/Parkin/PINK1 signaling pathway. The activation of parkin and PINK1 may be a potential mechanism of resveratrol for treating cardiovascular complications related to aging.

In their primary research article, K. Ji et al. evaluated ginger oleoresin, mainly comprised of phenolic gingerols and shogaols, for their potential to alleviate *γ*-ray irradiation-induced reactive oxygen species. Besides the direct radiation damage, most of the ionizing radiation- (IR-) induced injuries are caused by generation of reactive oxygen species (ROS). Thus, alleviation of such ROS represents an important means for radiation protection. Their results showed that ginger oleoresin exerted radioprotective effect via the induction of the translocation of Nrf2 to cell nucleus and the subsequent activation of the expression of cytoprotective genes encoding HO-1 and NQO-1, suggesting that ginger oleoresin has a potential as an effective antioxidant and radioprotective agent.

M. Strzemski et al., in their article, studied the chlorogenic acid content, mineral content, total phenolic content, total flavonoid content, and antioxidant activity of three populations of *Carlina vulgaris* L. Among these populations, the flower head extracts obtained from the nonmetallicolous populations were shown to contain the largest amount of chlorogenic acid, a major antioxidant component of this species. Their study suggested *Carlina vulgaris* L. as a source of phytochemicals with antioxidant activity.

The review article by Y. Zhao et al. summarized the recent experimental and limited clinical trial evidence supporting that quercetin, curcumin, and resveratrol, the three selected phenolic compounds, have potential beneficial functions on obesity treatment, through the alleviation of intracellular oxidative stress as one of the possible mechanisms.

A. Papadopoulou et al. observed the reduction of oxidative stress in chickens after the administration of drinking water rich in polyphenolic powder from olive mill waste waters. They evaluated the biomarker in plasma associated with the reduction of oxidative stress and suggested that the phenolic components from olive mill waste waters could be utilized as a supplement to reduce oxidative stress-induced damage in chicken raising.

In a primary research study, L. Du et al. showed that sea buckthorn paste, a traditional Tibetan medicine with high content of polyphenols and remarkable antioxidant activity, provided significant protection against LPS-induced acute lung injury through maintaining redox homeostasis, with the underlying mechanism involving Nrf2 nuclear translocation and activation.

H. Zhou et al., in their article, revealed that rosmarinic acid attenuated the endothelial dysfunction induced by oxidative stress via the activation of the AMPK/eNOS pathway. They showed that rosmarinic acid cotreatment mitigated the endothelium-dependent relaxation impairments and the oxidative stress induced by H_2_O_2_ and reversed the downregulation of AMPK and eNOS phosphorylation.

In another review article, L. Yu et al. summarized the recent research development with regard to the cardioprotective effects of salvianolic acid, the major bioactive phenolic constituent of Radix *Salviae miltiorrhizae*. They used the vascular endothelium growth factor, blood vessel density, and myocardial infarct size as the outcome measures for evaluation. Their meta-analysis demonstrated that salvianolic acid can exert cardioprotection effect through promoting angiogenesis in animal models of myocardial infarction.

The article of H. Wu et al. investigated the hepatoprotective effect and underlying mechanisms of the polyphenol-enriched fraction from *Folium Microcos*. Their findings suggested that the hepatoprotective effect of *Folium Microcos* against APAP-induced hepatotoxicity is mainly through dual modification of ROS/MAPKs/apoptosis axis and Nrf2-mediated antioxidant response, which might be attributed to the strong antioxidant activity of phenolic components.

*Jie Li* *Keyvan Dastmalchi* *Lin-sen Qing* *Pei Luo*

